# Electrochemical Analysis and In Vitro Assay of Mg-0.5Ca-xY Biodegradable Alloys

**DOI:** 10.3390/ma13143082

**Published:** 2020-07-10

**Authors:** Bogdan Istrate, Corneliu Munteanu, Stefan Lupescu, Romeu Chelariu, Maria Daniela Vlad, Petrică Vizureanu

**Affiliations:** 1 Mechanical Engineering Department, Gheorghe Asachi University of Iasi, 6 D. Mangeron Blvd, 700050 Iasi, Romania; bogdan_istrate1@yahoo.com; 2 Faculty of Material Science and Engineering Department, Gheorghe Asachi University of Iasi, 41 DimitrieMangeron str., 700050 Iasi, Romania; rchelariu@yahoo.com (R.C.); peviz2002@yahoo.com (P.V.); 3 Faculty of Medical Bioengineering, “Grigore T. Popa” University of Medicine and Pharmacy from Iasi, 9-13 Kogălniceanu Str, 700454 Iasi, Romania; maria.vlad@umfiasi.ro; 4 Romanian Inventors Forum, Sf. P. Movila 3, 700089 Iasi, Romania

**Keywords:** Mg-Ca-Y alloys, microstructure, electrochemical evaluation, in vitro test

## Abstract

In recent years, biodegradable Mg-based materials have been increasingly studied to be used in the medical industry and beyond. A way to improve biodegradability rate in sync with the healing process of the natural human bone is to alloy Mg with other biocompatible elements. The aim of this research was to improve biodegradability rate and biocompatibility of Mg-0.5Ca alloy through addition of Y in 0.5/1.0/1.5/2.0/3.0wt.%. To characterize the chemical composition and microstructure of experimental Mg alloys, scanning electron microscopy (SEM), energy-dispersive spectroscopy (EDS), light microscopy (LM), and X-ray diffraction (XRD) were used. The linear polarization resistance (LPR) method was used to calculate corrosion rate as a measure of biodegradability rate. The cytocompatibility was evaluated by MTT assay (3-(4,5-dimethylthiazole-2-yl)-2,5-diphenyltetrazolium bromide) and fluorescence microscopy. Depending on chemical composition, the dendritic α-Mg solid solution, as well as lamellar Mg_2_Ca and Mg_24_Y_5_ intermetallic compounds were found. The lower biodegradability rates were found for Mg-0.5Ca-2.0Y and Mg-0.5Ca-3.0Y which have correlated with values of cell viability. The addition of 2–3 wt.%Y in the Mg-0.5Ca alloy improved both the biodegradability rate and cytocompatibility behavior.

## 1. Introduction

Nowadays, because of an increasing number of humans having a diversity of diseases or traumas of skeletal system, there has been an increasing demand for osteosynthesis devices (plates, screws, prostheses, rods, implants, etc.) [1,2,3]. Some of these devices are dedicated to maintaining structural stability and aligning bone fragments for a finite time during the healing process of fractures, so that they became temporary devices [4]. Traditionally, such temporary osteosynthesis devices are made from inert metallic materials, but this approach requires a second surgical procedure to extract the device at the end of the healing process [1,2,3,4,5,6,7]. Rahim et al. [2] set some important requirements for degradable orthopedic implants to allow the healing of broken bones, like high biocompatibility, tissue friendly self-degrading, and minimal stress-shielding effects. Furthermore, recent research mentioned that the interference screws are made of an MgYREZr-alloy which has been introduced to the market (Milagro; DePuyMitek, Leeds, UK). Banerjee et al. [3] highlights the prospects of Mg alloy implants and various coatings due to their fast degradation in physiological fluids. Another approach in the fabrication of temporary osteosynthesis devices appeared with the development of body-absorbable polymers [1,5]. To eliminate the main drawback of body-absorbable polymers, the unacceptable decreasing of mechanical properties during the healing process [3], in the last two decades Mg-, Fe-, and Zn-based materials were intensively investigated as body-degradable materials [8], mainly Mg-based materials [2,3,5].

Mg is an important mineral in the human body, it has an important role for many physiological functions, and it is an essential mineral for bone formation [5,9,10]. The density, elastic modulus, compressive yield stress, and fracture toughness are closer to those of natural bone in comparison with those of inert metallic or ceramic biomaterials [5]. Mg corrodes in aqueous environments, which makes it a body-degradable material [5], but it has the drawback of degrading with a corrosion rate much higher than that required in temporary osteosynthesis applications, and this process led, among others, to the hydrogen evolution in a significant volume [2,3,5,11]. It is known that other elements such as impurities (Fe, Ni, Cu, etc.) are identified in Mg-based alloys. Their chemical concentration significantly influences the corrosion process. Thus, the studies performed by Atrens et al. [12] and Liu et al. [13] highlighted the major effects that metallic impurities have on Mg-based alloys. These elements form secondary phases and influence by at least an order of magnitude the degradation rate in specific solutions.

In order to correlate the corrosion rate to the osteosynthesis timeframe of Mg, in a quantity that can be considered to estimate body-degradability rate, some have used alloys with different metals, which can be a beneficial approach for mechanical properties as well [5,11,14]. Mg-rare earths (REs) alloys from binary, ternary, and quaternary system alloys were investigated regarding the mechanical properties and the corrosion behavior, as well as from the biocompatibility point of view [15]. Although an improvement of both the mechanical properties and the corrosion behavior for an appreciable number of alloys was attained, the biocompatibility did not follow the same trend [15], controversial experimental data being reported about effects and toxicity of REs [15,16,17,18,19]. Because REs are mixtures of some lanthanides in various chemical compositions, a way to better control the effects of such elements on the biocompatibility of Mg-REs alloys is to use only one of the elements as alloying element in biodegradable Mg alloys [16]. From REs group besides lanthanides there are Y and Sc [16] which can have similar effects on the microstructure and properties of Mg alloys with those of lanthanide mixtures [20,21,22,23,24,25,26]. Adding of Y significantly improves the mechanical properties in monolithic Mg, while Sc increases corrosion resistance. Liu et al. [27] divided the rare earth elements into two main categories: I (Sc, Nd, Sm, Eu, Gd, Tb, Tm, and Yb) with applications in the cardiovascular field (stents) and II (Y, La, Ce, and Pr) for orthopedic applications (biodegradable implants). Y presents a maximum solubility of 4.7 at.%(15.28 wt.%) in Mg and it strengthens the solid solution. Similar aspects of increasing mechanical strength and ductility were observed for Mg-2.0Y and Mg-3.0Y [20]. Tekumalla et al. also reported that Y highlights a negative aspect on Mg corrosion resistance and Mg-1Y presented very low toxicity to osteoblast cells.

Ca has a critical role for a broad range of physiological functions and it is the most abundant mineral in the human body [28,29,30,31]. Ca in Mg alloys has the role of grain refiner that leads to an improvement of ultimate tensile strength and creep resistance [32]. Binary Mg-Ca alloys have been investigated for orthopedic applications and it has been found that Mg-Ca alloys with a Ca content up to 1 wt.% are suitable to make degradable implants [32,33,34,35,36,37,38,39,40,41,42,43,44,45,46,47,48,49,50], the higher contents affecting the castability [32], the corrosion behavior, and mechanical properties due to the higher volume fraction of Mg_2_Ca phase [33,34,35,38,39], and the biocompatibility [35,36].

The cellular viability evaluation using L-929 cells presented by Li et al. [35] showed that Mg-1Ca alloy did not induce toxicity to cells. A 1 wt.% Ca in Mg alloy presented high activity of osteoblasts and osteocytes due to the implanted pin into the femoral shafts of a rabbit, respectively, for 1, 2 and 3 months. Also, Erdmann et al. [41] and Zeng et al. [48] showed that Mg-0.8Ca and Mg–0.79Ca alloys have good cellular response and high mechanical resistance (hardness, ultimate tensile strength, and yielding strength) for up to six weeks during in vivo implantation. After this period, a gradual degradation was observed.

The properties of the Mg-Ca alloys are influenced by mechanical or thermo-mechanical processing [39,41,42,43,46,47,48,49,50]. However, e.g., Mg0.8Ca alloy tested in vivo has shown an insufficient initial strength and a fast degradation [40]. Thus, alloying the Mg-Ca alloys (Ca wt.% < 1) with a third element, e.g., from REs group, can be a way to improve the properties and biocompatibility.

In this paper, a Mg-0.5Ca alloy was alloyed witha content of 0.5, 1.0, 1.5, 2.0, and 3.0 wt.%Y to improve the degradability. Additionally, the biocompatibility of the studied alloys was tested in vitro.

## 2. Materials and Methods

### 2.1. Synthesis of Mg-Ca-Y Alloys, Morphological and Structural Analysis

The master alloys used for sample manufacturing were purchased from Hunan China Co., Hunan, China [51,52] and they have the chemical composition presented in Table 1. The casting of Mg alloys was performed in an induction furnace (Inductro S.A., Bucharest, Romania) with an inert Ar5.0 protective atmosphere at a temperature of 680–690 °C for 30 min using rectangular bars as raw materials. The resulting mini-ingots were cut into spherical samples having different concentrations as is presented in Table 2, with a diameter of 20 mm and a thickness of 2 mm. The amount of metallic charge for the experimental samples is presented in Table 2 and is about 23 g/ingot. The samples were grounded with abrasive discs with granulation between 340–2000MPi, polished with alumina suspension (1 µm–6 µm), cleaned with alcohol, and then ultrasonically cleaned in ethyl alcohol for 10 min. The experimental samples were etched with Mg(CH_3_COO)_2_*4H_2_O acetate solution for microstructural analysis. Surface morphology was investigated by light microscopy (Leica DMI 5000 microscope, Wetzlar, Germany) and scanning electron microscopy (SEM FEI Quanta 200 3D, dual beam, equipped with energy dispersive X-Ray spectroscopy analysis unit—Xflash Bruker, Harvard, MA, USA). X-Ray diffractions (XRD, Panalytical, Almelo, The Netherlands) were performed using a Xpert PRO MPD 3060 facility from Panalytical (Almelo, The Netherlands), with a Cu X-ray tube (Kα = 1.54051Å), 2 theta: 30°–100°, step size: 0.13°, time/step: 51 s, and a scan speed of 0.065651°/s.

### 2.2. Electrochemical Analysis

Mg alloys were tested in a simulated body fluid (SBF) with the chemical composition presented in Table 3, according to the procedure described in [53]. Potential measurements were performed using a VoltaLab 21 potentiostat (Radiometer Analytical SAS, Lyon, France). Data acquisition and processing was performed with Volta Master 4 software. A three-electrode cell was used: a platinum auxiliary electrode, a calomel saturated electrode, and a work electrode, with the specification that the working electrodes (samples) were removed from the resin and polished in parallelepiped forms. The experimental samples were mounted in Teflon support to allow connection to the electrochemical system electrode. The representation of linear polarization curves in the following coordinates: current density (mA/cm²) versus potential (V), allowed the highlighting of the corrosion potential (E_cor_), and the corrosion currents (J_cor_). Measurements were made at 25 °C and the electrolyte was naturally aerated, the linear polarization curves were recorded at a scanning potential of 1mV of the electrode and the cyclic polarization curves were performed at a scanning speed of 10mV.

The instantaneous corrosion rate, V_cor_ (mm/y), was determined from the corrosion current density, J_cor_ (mA/cm^2^) [22]:

V_cor_ = 22.85 × J_cor_, (1)

### 2.3. Cytocompatibility Testing

#### 2.3.1. Alloy Sample Preparation

Samples in the form of flat metal pieces (weighing between 0.68 g and 0.98 g) were cleaned with acetone–ethanol by sonication and exposed for 30 min on each side to the ultraviolet (UV) action for sterilization, and subsequently placed in hanging cell culture inserts (with a pore size of 0.4 μm) used in a 24-well plates to coincubate the alloy samples with the cells for the cell viability study. In addition, the studied Mg-0.5Ca-xY alloy samples were immersed in complete culture media, at 37 °C and 5% CO_2_, over a period of 5 days, for evaluating the pH variation during the co-incubation times (1, 3, and 5 days).

#### 2.3.2. Cell Culture

Albino rabbit dermal primary fibroblasts at passage 3 were selected for the viability study. The cells were cultured in DMEM-F12 Ham culture medium (Dulbecco’s modified Eagle medium/Nutrient F-12 Ham) supplemented with 10% fetal bovine serum (FBS) and 1% antibiotic (penicillin–streptomycin–neomycin) in humidified atmosphere of 95% air, 5% CO_2_ at 37 °C. The culture medium was changed every 2 days.

Cultures of 90% confluent cells were rinsed with prewarmed phosphate buffered saline (PBS) and harvested by incubating with trypsin/EDTA (Sigma Chemical Co., Saint Louis, MO, USA). Afterwards, the cells were detached, suspended in fresh media again, counted using a Neubauer counting chamber, seeded in 24 well culture plates at a density of 1 × 10^4^ cells/well/0.5 mL complete DMEM-F12, and incubated under the above mentioned conditions for 24 h to facilitate cell adhesion. Then, the medium was removed by aspiration, the cells were washed with PBS three times to eliminate unattached or dead cells and the inserts containing the studied alloy samples were placed on the assigned wells containing the cells cultured for 24 h. In addition, wells containing cell culture without alloy samples were used as controls (control-wells, i.e., negative control). It should be mentioned that the alloys’ interaction/reaction with the cell culture media took place during the cell culture test. Coincubation of the metal samples (3 samples for each alloy) with the cells was performed for 1, 3, and 5 days respectively for both the cytocompatibility testing and cell morphology study.

#### 2.3.3. Cell Viability

Cell viability was tested by the MTT assay (3-(4,5-dimethylthiazole-2-yl)- 2,5-diphenyltetrazolium bromide), which allows quantification of a metabolic activity causally related to the live cell [54]. For this purpose, following the incubation period, inserts were removed from the wells, the cells were rinsed with PBS and then MTT dye solution dissolved in fresh medium was added to the cells for 3 h at 37 °C in order to assure formation of the intracellular formazan crystals [55,56]. Subsequently dark-blue insoluble product formed inside the viable cells was solubilized with isopropyl alcohol under continuous agitation (Environmental Shaker-Incubator ES-20, Biosan, Riga, Latvia) for 15 min. The liquid of each sample was removed for the assay, which was performed in a 96-well plate, on a microplate reader (Tecan Sunrise, with Magelan V.7.1 soft for data acquisition, Tecan Group Ltd., Männedorf, Switzerland) at a wavelength of 570 nm.

Cell viability was expressed as a percentage related to the control wells, according to the formula CV = 100 × (ODs-ODb)/(ODc-ODb) where ODs represents the optical density for the sample wells (i.e., wells containing cell culture coincubated with alloy samples), ODb—optical density of the wells without cells or medium (blank), and ODc—optical density for the control-wells (i.e., wells containing cell culture without alloy samples).

For statistical analysis of cell viability results, the ANOVA one-way test was used and the data were compared using Tukey’s method, the statistically significant difference being accepted for *p* < 0.05.

#### 2.3.4. Cell Morphology

The cell morphology study was performed, at specified time periods of 1 day and 5 days, by fluorescence microscopy. For this, the cells were washed with Hanks’ Balanced Salt solution (HBSS; H8264, Sigma–Aldrich, Taufkirchen, Germany) without red phenol, and 200 μL of a 1:1000 calcein solution (Calcein AM; C1359, Sigma–Aldrich, Taufkirchen, Germany) in HBSS was added to each well and the plate was incubated in the dark for 30 min at 37 °C. Subsequently, cellular morphology was assessed by an inverted microscope (Leica DMIL LED equipped with camera Leica DFC450C and soft Leica Application Suite, Version 7.4.1 for image acquisition, Leica Microsystems, Wetzlar, Germany).

## 3. Results and Discussions

The microstructure of the synthesized alloys was presented through the experimental results. Through microscopy both the microstructure of new Mg-based alloys and the state of the surface before and after the tests of corrosion resistance were analyzed. The chemical compounds formed on the surface were determined and were followed by the cellular viability testing by MTT assay.

### 3.1. Structural Characterization

**Figure 1:**
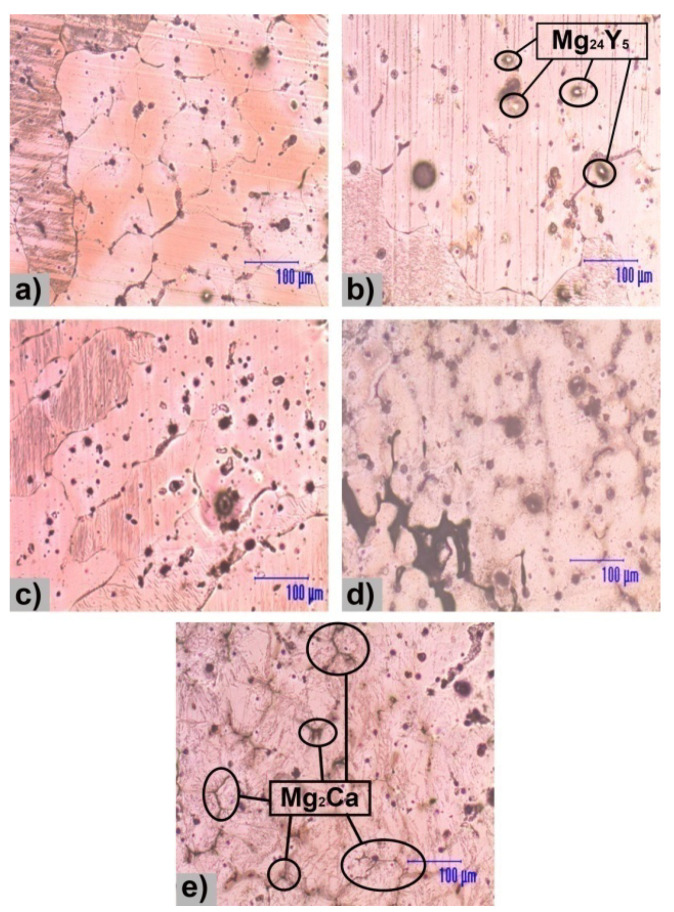
Light microscopy analysis of the Mg-0.5Ca-xY: (**a**) Mg-0.5Ca-0.5Y; (**b**) Mg-0.5Ca-1.0Y [57]; (**c**) Mg-0.5Ca-1.5Y; (**d**) Mg-0.5Ca-2.0Y [57]; (**e**) Mg-0.5Ca-3.0Y [57].

**Figure 2:**
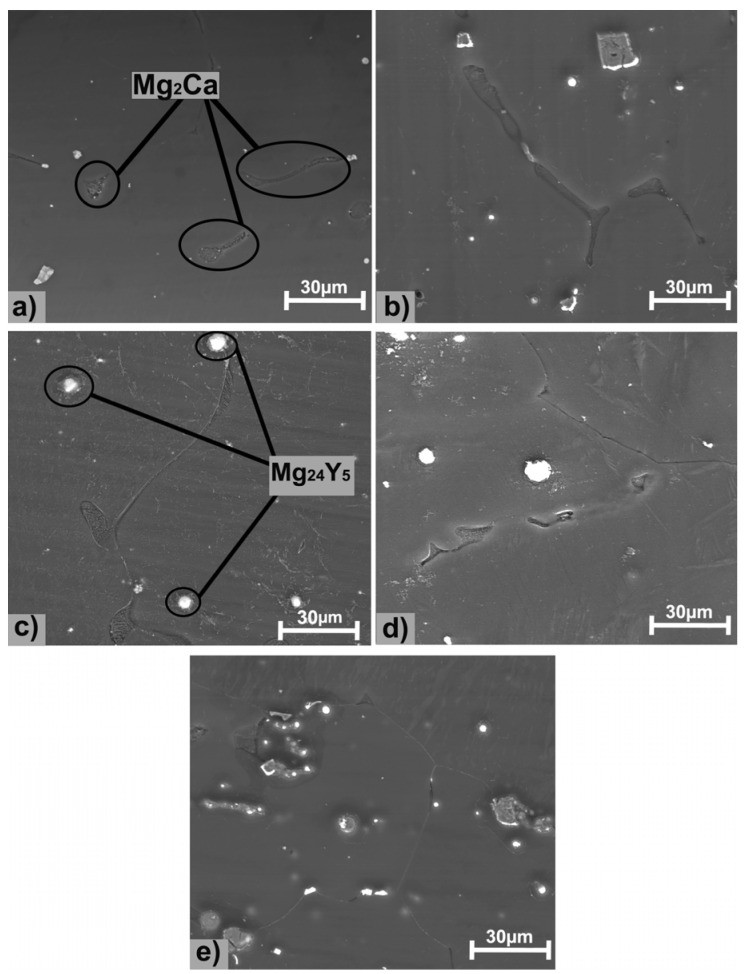
Surface SEM images of the Mg-0.5Ca-xY: (**a**) Mg-0.5Ca-0.5Y; (**b**) Mg-0.5Ca-1.0Y [57]; (**c**) Mg-0.5Ca-1.5Y; (**d**) Mg-0.5Ca-2.0Y [57]; (**e**) Mg-0.5Ca-3.0Y [57].

The structural characterizations of the five Mg alloys by microscopy are shown in Figure 1. The structure presents specific morphology of as-cast metallic materials, Mg_2_Ca lamellar intermetallic compounds and relatively uniform presence of Y-containing particles. The addition of Y led to the formation of globular particles with a segregating tendency of relatively uniform color [26]. The morphological aspect of the experimental samples is presented in Figure 2, where the presence of an intermetallic phase is observed. Y-containing particles are typically of about 15 µm and appear in a brighter contrast than Mg_2_Ca lamellar intermetallic compounds. The surface morphological investigations of the present study add new information to some previous researches conducted by Istrate et al. [57] in the case of Mg-based alloys having a Y concentration between 1.0 wt.% and 3.0 wt.%.

The chemical composition of the Mg-0.5Ca-xY alloys was investigated by Energy-Dispersive X-ray Spectroscopy analysis. Transverse sections of the samples were used for the examination in ten different areas and the average results are presented in Table 4.

**Figure 3:**
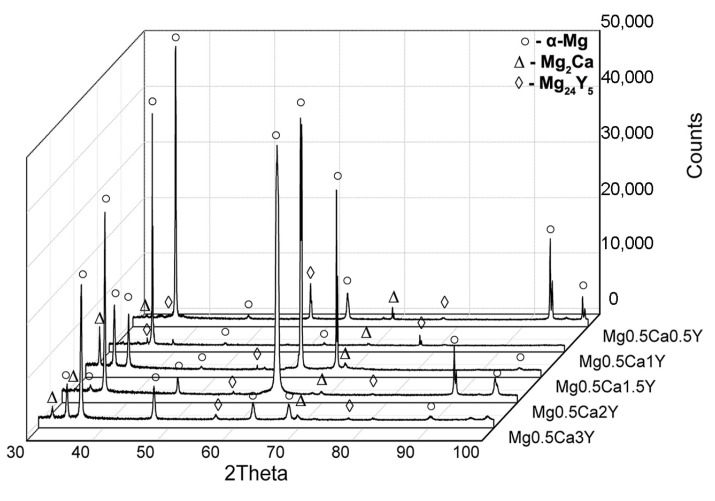
XRD analysis of Mg-0.5Ca-xY experimental alloys [57].

Microstructural analysis highlights homogeneous structures with the formation of specific phases, namely α-Mg-based solid solution (α-Mg), Mg_2_Ca, and Mg_24_Y_5_. The Mg_2_Ca compound is located at the boundaries of the α-Mg grains, forming an eutectic compound with α-Mg. The XRD patterns for the Mg-0.5Ca-xY are presented in Figure 3. α-Mg (ICDD-PDF: 01-071-9399) has been identified at 2θ = 36.54°, 47.69°, 57.28°, 68.49°, and 90.21°, as predominant phase having a hexagonal crystalline structure. Furthermore, the presence of Mg_2_Ca (ICDD-PDF: 01-073-5122) was revealed at 2θ = 34.11°, 52.35°, and 69.12° and Mg_24_Y_5_ (ICDD-PDF: 01-071-9618) in cubic form at 2θ = 37.48°, 57.02° and 75.78°.

### 3.2. Electrochemical Evaluation

The main parameters of the corrosion process performed in SBF solution are presented in Table 5. Since Mg alloys eliminate high amounts of gas on the surface of the samples, gas bubbles are constantly formed. These gas bubbles were removed by using a magnetic stirrer that operated at a relatively slow rate of agitation of the electrolyte solution. At each test, the fresh electrolyte solution was used. The corrosion current (J_cor_) shows the degradation degree of the experimental alloys. It revealed different values of corrosion density between 0.3663 mA/cm² and 2.9812 mA/cm² for all the samples.

**Figure 4:**
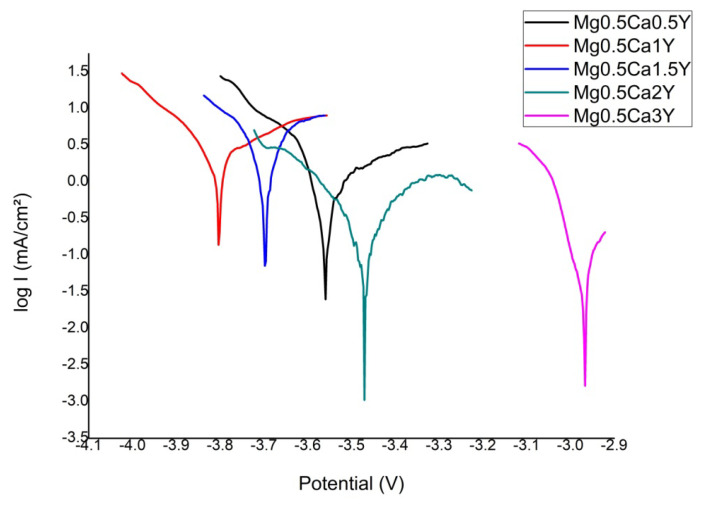
Tafel diagrams of Mg-0.5Ca-xY-based experimental alloys.

**Figure 5:**
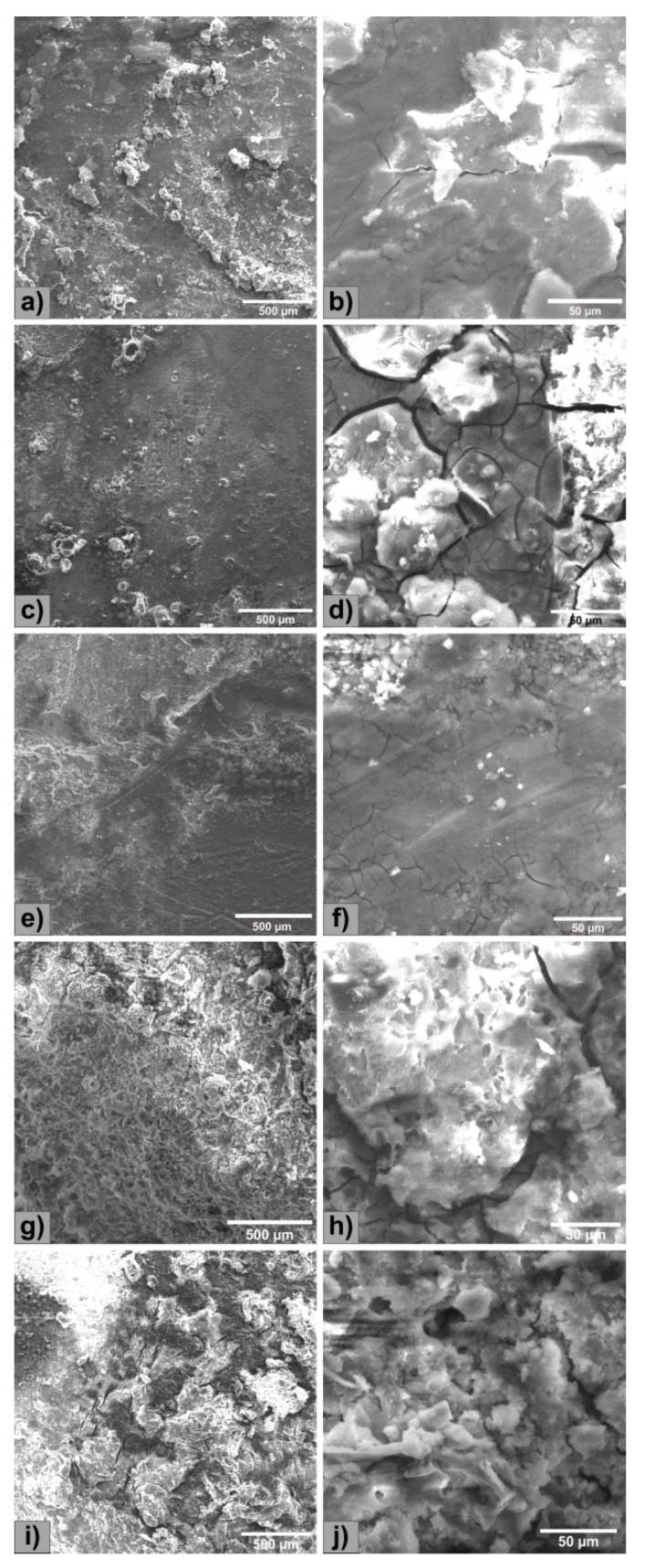
SEM images of experimental surfaces after electrochemical tests: (**a**,**b**) Mg-0.5Ca-0.5Y; (**c**,**d**) Mg-0.5Ca-1.0Y; (**e**,**f**) Mg-0.5Ca-1.5Y; (**g**,**h**) Mg-0.5Ca-2.0Y; and (**i**,**j**) Mg-0.5Ca-3.0Y.

To confirm the effect of the polarization (Figure 4) and to understand the electrochemical corrosion mechanism of the Mg-0.5Ca-xY alloys, surface morphology was investigated by SEM (Figure 5). The purity of Mg is 99 wt.%, the rest of the elements being accompanying elements, used in the synthesizing process, which led to the obtaining of experimental alloys with some impurities (Fe, Ni, Cu, Si). The alloying elements contribute to the formation of additional phases in the α-Mg matrix (Mg_2_Ca and Mg_24_Y_5_).

The analysis of the experimental results reveals the effect of the alloying element Y on the electro-corrosion resistance properties, namely a reduction of the corrosion rate of up to 6.68 times with the addition of 3.0 wt.% of Y or 8.14 or for the addition of a percentage of at least 2.0 wt.%Y. From the point of view of corrosion resistance, the percentage of Y significantly influences the behavior of the alloy to values higher than 1.5 wt.%Y. It is known that the both intermetallic compounds significantly influence the corrosion behavior of Mg alloys [23,24]. Mg_2_Ca suffers of dissolution at high rate [24], and when the fraction of Mg_24_Y_5_ increases, the corrosion current density increases [23]. Also, although Y has a limited solubility in Mg, up to a certain content of Y dissolved in Mg improve corrosion behavior of Mg matrix. Having in view those previously mentioned, at low content of Y (up to 1.0 wt.%) the Mg_2_Ca and Mg matrix lead to high values of J_cor_. When content of Y increases (up to 2.0 wt.%) the dissolution rate of Mg matrix decreases. A supplementary increase of Y content (up to 3.0 wt.%) causes an increase of the Mg_24_Y_5_ fraction which leads to a slight increasing of J_cor_ values. Also, the impurities could either form specific compounds or segregate at grain boundary, what can significantly accelerate corrosion by micro-galvanic corrosion. This can lead to higher degradation rates [58], but, as the diffraction patterns show, probably these phases are in extremely low fraction because all detectable peaks of the XRD patterns were assigned to α−Mg, Mg_2_Ca, and Mg_24_Y_5_.

All samples exhibit a corroded surface, especially due to the potentiodynamic polarization test, the corrosion being of a generalized type, and in all cases the formation of surface compounds occurs by the interaction of the alloy and the electrolyte solution. The corrosion products formed on the surface of Mg alloys exhibit numerous cracks and have different morphological aspects.

**Figure 6:**
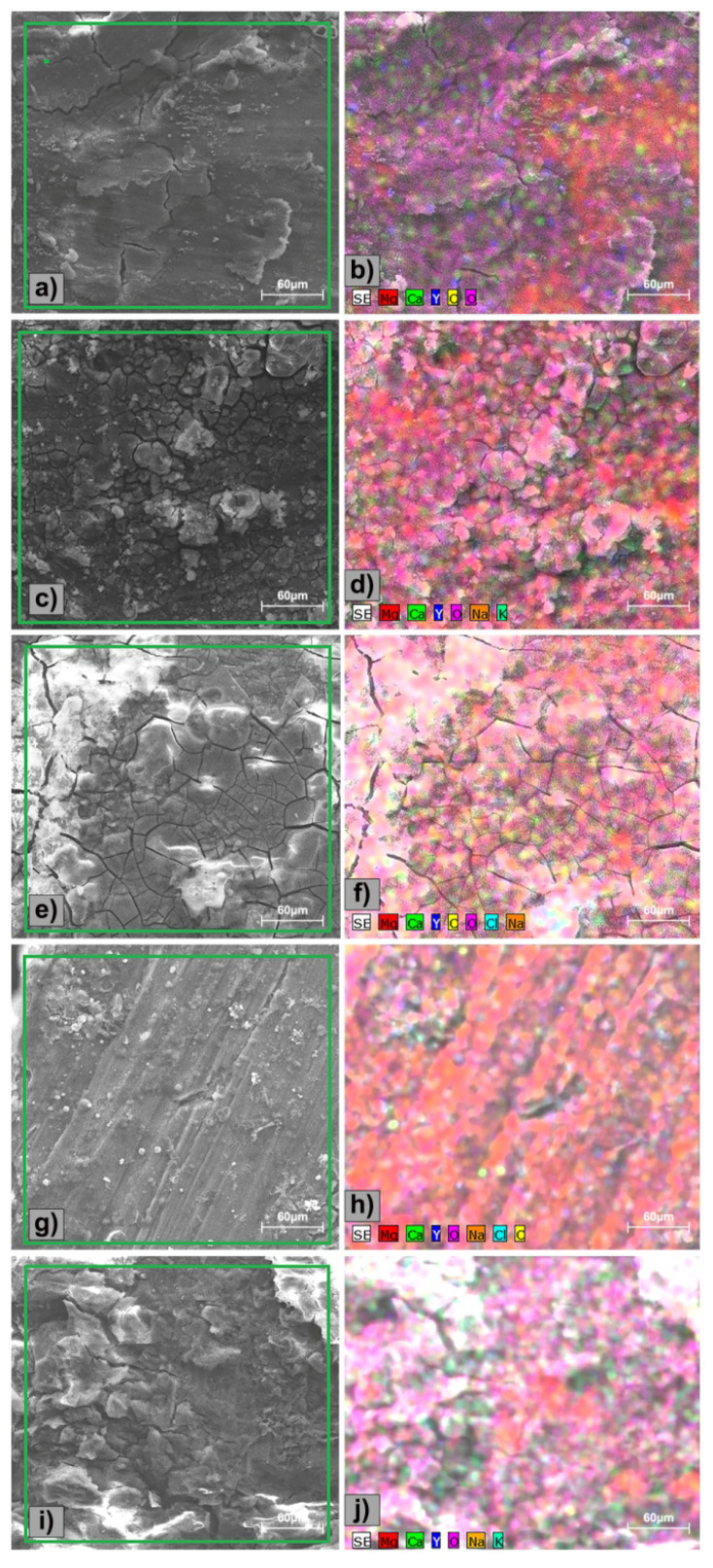
Distribution of the identified elements on the surface of the Mg-0.5Ca-xY alloys after electrochemical tests: (**a**,**b**) Mg-0.5Ca-0.5Y; (**c**,**d**) Mg-0.5Ca-1.0Y; (**e**,**f**) Mg-0.5Ca-1.5Y; (**g**,**h**) Mg-0.5Ca-2.0Y; and (**i**,**j**) Mg-0.5Ca-3.0Y.

The quantitative determination of the elements existing on the surface of the alloy led to the results shown in Table 6. It can be considered that due to the alloying elements there is availability for the formation of oxides and hydroxides by the reaction with the electrolytic SBF medium.It can be observed that the chemical composition of the sample surface is influenced by the behavior of the Y element on the Mg alloy which is close to that of the original material. The difference is being attributed to the standard EDS detector’s error and also to the partial oxidation of the Y-based compounds. The distribution of the elements identified on the Mg-0.5Ca-xY alloy surface after the electrochemical corrosion test was investigated by SEM-EDS (Figure 6). A higher percentage of Y over 1.5 wt.% is a success in terms of corrosion resistance, an affirmation confirmed by the linear polarization results. According to references [22] and [37], Atrens et al. and Li et al. showed the formation of Mg-Y and Mg-Ca compounds after the electrocorrosion process. The results of the present research highlight that the entire surface of the samples is covered by O and Mg compounds. The Y and Ca elements exhibit a similar behavior to the other experimental alloys [22,37], by forming rich-Y precipitates (Mg_24_Y_5_) and Mg_2_Ca compound at the grains boundary.

In the case of the 2.0 wt.%Y alloy, the presence of O, Na, C, and Cl elements was identified on the surface by the interaction of the experimental alloy with the SBF electrolyte solution. The presence of chlorine-based salts is also noted. On the surface of the Mg-0.5Ca-3.0Y alloy, in addition to the basic elements of the experimental alloy, the elements: O, K, and Na were also identified, mainly due to the interaction with the SBF.

### 3.3. Cytocompatibility Study

**Figure 7:**
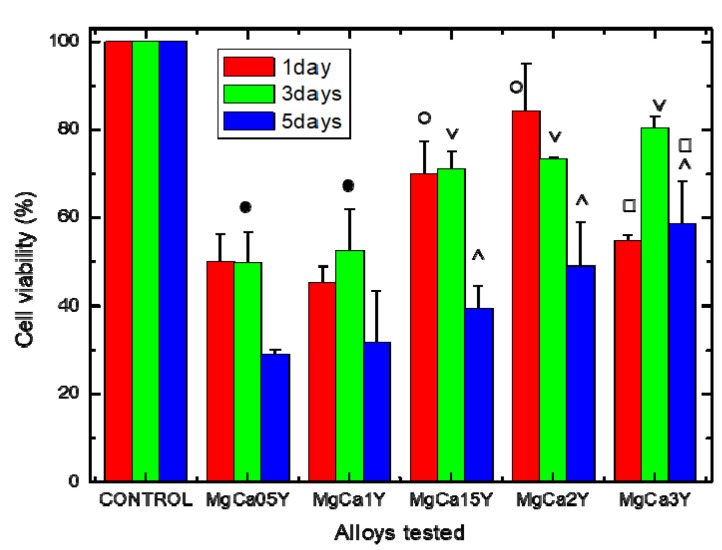
Cell viability profile (%) evaluated by 3-(4,5-dimethylthiazole-2-yl)- 2,5-diphenyltetrazolium bromide - (MTT)-assay: Effect of Mg-0.5Ca-xY experimental alloys on cell viability after 1, 3, and 5 days of co-incubation. Data expressed as percent related to the negative control. (˄; o; ●; ˅; □) No significant differences on cell viability (*p* > 0.05); see details in the text.

Figure 7 shows the results of the MTT assay for testing the cytocompatibility of the studied Mg-0.5Ca-xY experimental alloys, expressed as percentages of the control-wells’ viability (i.e., negative control). After 1 day of coincubation of the cells with the studied samples, cell viability profile was significantly higher in the case of alloys containing 1.5 wt.%Y and 2.0 wt.%Y respectively compared to the other studied alloys (*p* < 0.05). Cell viability profile after 3 days of coincubation was significantly lower in the case of alloys containing 0.5 wt.%Y and 1.0 wt.%Y respectively, compared to the other studied alloys (*p* < 0.05), while cell viability was not significantly different (*p* > 0.05) for alloys with a Y content of 1.5 wt.%, 2.0 wt.%, and 3.0 wt.%, respectively. In addition, it was observed that the cells’ viability profile decreased significantly with the time, after 5 days being lower than for the other time periods (*p* < 0.05), and appearing to be inversely proportional to the amount of Y used for alloying. However, cell viability after 5 days was not significantly different (*p* > 0.05) for alloys with Y amount of 1.5 wt.%, 2.0 wt.%, and 3.0 wt.%, respectively. In the case of the alloy obtained by alloying with 3.0 wt.% Y the cell viability level was not significantly different (*p* > 0.05) after 5 days comparing with those obtained after 1 day of incubation.

**Figure 8:**
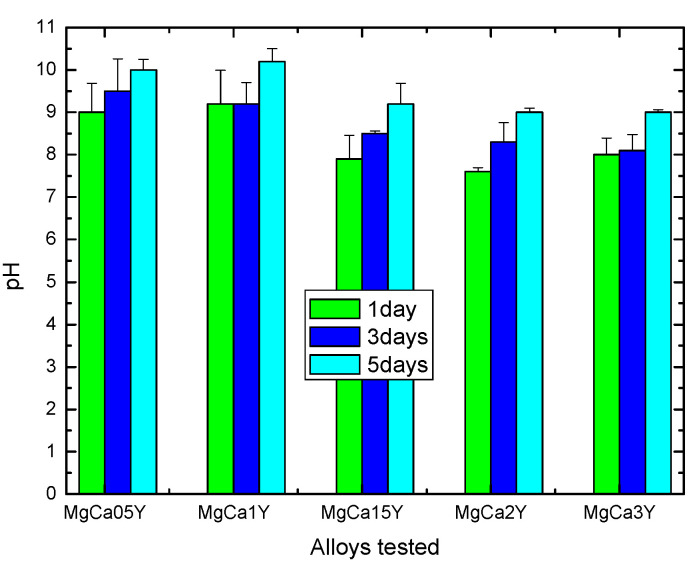
Variation of the pH in the DMEM-F12 complete media (Dulbecco’s modified Eagle medium/Nutrient F-12 Ham) during co-incubation with the studied Mg-0.5Ca-xY alloys samples (at 37 °C, 5% CO_2_), over a period of 5 days.

The results of the cytocompatibility study seem to suggest that by increasing the amount of Y used for alloying, cytocompatibility was improved and this was probably due tothe increase of the experimental alloys stability in humid environment having a complex composition (such as culture medium or biological fluids). The lower viability level compared with the control-wells (i.e., negative control) could be attributed to alloy reactivity at the contact with the culture medium [59], resulting in H_2_ release and immediate pH changes (i.e., subsequent alkalinization) of the cell culture medium (see Figure 8). It is known that the environment has a considerable influence on the degradation behavior of degradable materials [12], and consequently, the degradation of the Mg-alloys (accompanied by ions and degradation products release) is responsible for pH and osmolarity increasing, and this might adversely affect cells’ metabolic activity. In this sense, the increase of pH value after Mg-alloy immersion in aqueous environment is mainly the result of an anodic reaction of Mg^2+^ by reduction of H_2_O (to H_2_ gas and OH^−^) and production of Mg(OH)_2_ corrosion product.

However, the viability level both at 1 day and 3 days (≥70%) for alloys with Y content of 1.5 wt.%, 2.0 wt.% allows us to appreciate (based on ISO 10993-5, [60]) that the two alloys don’t have a cytotoxic effect, and this is so because of the decision to carry out the cytotoxicity tests by direct co-incubation of the studied alloys with the cells using hanging inserts (by which the alloys were suspended in the wells, in order to avoid cell damage by mechanical effect as a result of alloys samples’ disintegration). Consequently, by this way, the cells were continuously exposed (up to 5 days) to an 100% extract and, furthermore, continuously exposed to the sum of the phenomena that take place during Mg-alloys degradation, like hydrogen, ions, and degradation products release.

In this sense, a non-cytotoxic character could also be attributed to the alloy containing 3.0 wt.%Y, excepting the lower viability for this alloy at one day, possibly due to a higher reactivity, being a potential signal that by alloying with Y, an optimum is reached for 1.5 and 2.0 wt.%Y amounts. Indeed, this observation is sustained by other complementary data, such as corrosion resistance tests (see the above section).

Cell viability after 5 days decreased at values around 50%, especially for alloys with Y content of 1.5, 2.0, and 3.0 wt.% (and below 50% for other alloys), and these findings could be attributed to many factors such as modification of the pH values (as is shown in Figure 8) that may affect cell metabolic activity, ionic massive release from alloys, and precipitation of salts with inhibitory or toxic effect, the increase of osmolarity as a consequence of reactivity that may lead to hyperosmotic shock [61]. It is true that any inhibitory or cytotoxic effect is of concern regarding the potential in vivo cytotoxicity, but only the in vitro cytotoxicity data not essentially discard the biomaterials’ potential use in clinical application [60], because of different real corrosion/degradation scenario of Mg-alloys under biomedical applications (i.e., both composition and flow of biological fluids) that may affect the local surface chemistry of the implants.

**Figure 9:**
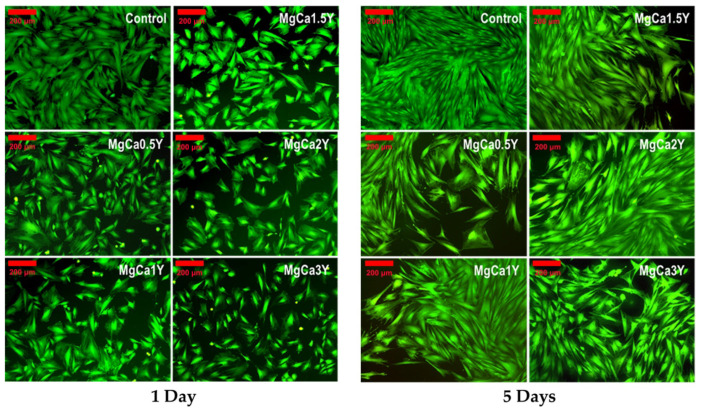
Fluorescence microscopy images of fibroblastic cells’ morphology after 1 and 5 days of coincubation with the experimental alloys. Viable cells stained in green because of calcein fluorescent dye presence inside the cells. Bar: 200 μm.

The cytocompatibility data were correlated with the cell density and morphology results (Figure 9) obtained for the studied alloys after 1 day and 5 days of coincubation. In this sense, cell’ density after 1 day was lower than that of the controlwells, which agrees with the cell viability data and this observation is maintained after 5 days of coincubation.

Figure 9 shows the observation performed by fluorescence microscopy on fibroblast cells density and morphology, coincubated with the studied alloys, for 1 day and 5 days. It is observed that after 1 day the cell density evidenced for the alloys is lower than that of the control-wells (i.e., negative control, containing only cells without metallic samples). Moreover, different cell morphology is observed, in the sense of an elongated aspect with bipolar morphology in areas with greater cell density, and a polygonal morphology with wide lamellipodia cytoplasmic processes in areas with low cell density. Furthermore, polygonal morphology was observed towards the center of the well and in the immediate surrounding area of the insert membrane where cell density was lower. Different cell morphology can also be attributed to different concentrations of Ca, Mg, and Y ions in the well microenvironment, knowing that Ca is associated with a structural role in the reorganization of cytoskeleton elements [62], whereas the accumulation of Mg ions over a certain limit may have an inhibitory effect [63]. However, it should be noted that all of the physico-chemical processes taking place during the degradation process of Mg alloys depend on the microenvironment conditions [64] and, from this point of view, under amicroenvironment governed by active transport processes (as is the case of the in vivo degradation), these physico-chemical phenomena may be very well balanced and the results of in vivo biodegradation and osteointegration are somehow optimal. Accordingly, in order to prove the biocompatible behaviour of the studied Mg-alloys a pending in vivo study is expected to clarify this concern.

## 4. Conclusions

In the present study, five differently prepared biodegradable Mg alloys, Mg-0.5Ca-0.5Y, Mg-0.5Ca-1.0Y, Mg-0.5Ca-1.5Y, Mg-0.5Ca-2.0Y, and Mg-0.5Ca-3.0Y, were investigated. The results can be concluded as follows:

(1) Addition of Y in the experimental alloys refines the microstructure, resulting in the Mg_24_Y_5_ cubic structure compound. Y compounds have a white spherical form in the metallic matrix andtypically a size of 15 µm. Also, Ca forms an eutectic compound—Mg_2_Ca, founded at the Mg grainsboundary.
(2) The corrosion resistance was performed in SBF solution and presented a generalized type with very few areas not affected by corrosion. Addition of Y leads to an increase in electro-corrosion resistance, especially at alloying percentages greater than 1.0 wt.%. Increasing the content of Y, the immersion and electro-chemical tests show an improved degradation rate as following: 65.7 mm/y (0.5 wt.%) and 10.20 mm/y (3.0 wt.%).
(3) The Mg-0.5Ca-xY alloys have a cytocompatible behavior, i.e., the viability level at 3 days for Mg-Ca-Y alloys with a Y amount of 1.5, 2.0 and 3.0 wt.% was above 70%. The decrease of cell viability level after 5 days at values around 50%, especially in the case of alloys with 1.5 wt.%, 2.0 wt.% and 3.0 wt.% Y, should be attributed to the following factors: change of pH value, ion release from alloys, increasing of osmolarity, and salt precipitation with toxic or inhibitory effect. However, the increase of the Y alloying amount seems to increase the cytocompatibility of these alloys and open the way for future studies concerning alloys with a content higher than 3.0 wt.% Y.

## Figures and Tables

**Table 1:** Chemical composition of Mg master alloy—Hunan China Co. [51,52].

Alloys	Mg/Ca/Y (wt.%)	Fe (wt.%)	Ni (wt.%)	Cu (wt.%)	Si (wt.%)	Al (wt.%)
Pure Mg	Mg (99 wt.%)	0.15–0.2	0.17–0.2	0.14–0.2	0.15–0.2	0.16–0.2
Mg15Ca	Ca (15.29 wt.%)	0.004	0.001	0.003	0.013	0.011
Mg30Y	Y (28.05 wt.%)	0.010	0.001	0.001	0.006	0.011

**Table 2:** Correspondence of codes of studied materials and the amount of metallic charge for the experimental alloys.

Sample Code	Mg (g)	Mg-15Ca (g)	Mg-30Y (g)
Mg-0.5Ca-0.5Y	21.82	0.77	0.41
Mg-0.5Ca-1.0Y	21.42	0.77	0.82
Mg-0.5Ca-1.5Y	21.00	0.77	1.23
Mg-0.5Ca-2.0Y	20.59	0.77	1.64
Mg-0.5Ca-3.0Y	19.77	0.77	2.46

**Table 3:** Chemical composition of immersion solution.

Chemical Composition (Ions) (mmol/dm^3^)	Na^+^	K^+^	Mg^2+^	Ca^2+^	Cl^−^	HCO_3_^−^	HPO_4_^2−^	SO_4_^2−^
Simulated body fluid	142	5	1.5	2.5	147.8	4.2	1	0.5
Human blood plasma	142	5	1.5	2.5	103	27	1	0.5

**Table 4:** Average elemental compositions obtained by Energy-Dispersive X-ray Spectroscopy analysis.

Alloy		Mg (wt.%)	Ca (wt.%)	Y (wt.%)	Si (wt.%)	Fe (wt.%)	Ni (wt.%)	Cu (wt.%)
Mg-0.5Ca-0.5Y	Average	97.8	0.7	0.5	0.2	0.3	0.2	0.3
	Stdev	±0.4	±0.1	±0.1	±0.1	±0.1	±0.1	±0.1
Mg-0.5Ca-1.0Y	Average	97.3	0.6	0.9	0.2	0.3	0.3	0.3
	Stdev	±0.5	±0.1	±0.3	±0.1	±0.1	±0.1	±0.2
Mg-0.5Ca-1.5Y	Average	96.6	0.7	1.3	0.3	0.3	0.3	0.2
	Stdev	±0.2	±0.1	±0.1	±0.1	±0.1	±0.1	±0.1
Mg-0.5Ca-2.0Y	Average	96.2	0.7	1.9	0.2	0.3	0.4	0.4
	Stdev	±0.5	±0.1	±0.3	±0.1	±0.1	±0.2	±0.2
Mg-0.5Ca-3.0Y	Average	95.6	0.7	2.8	0.1	0.2	0.2	0.3
	Stdev	±0.4	±0.1	±0.1	±0.1	±0.1	±0.1	±0.2

Average elemental composition with calculated standard deviation on 10 analyzed surfaces per sample.

**Table 5:** Parameters obtained from the electro-corrosion resistance tests of the experimental alloys Mg0.5Cax (x = 0.5; 1.0; 1.5; 2.0; and 3.0 wt.%) Y.

Sample	E_0_	b_a_ (mV)	b_c_ (mV)	R_p_ (ohm/cm²)	J_cor_ (mA/cm²)	V_cor_ (mm/y)
Mg-0.5Ca-0.5Y	−3562.1	73.7	−40.9	49.99	2.8753	65.70
Mg-0.5Ca-1.0Y	−3801.9	479.8	−237.2	13.70	2.9812	68.12
Mg-0.5Ca-1.5Y	−3697.5	112.1	−102.8	12.95	1.5805	36.11
Mg-0.5Ca-2.0Y	−3470.8	269.3	−205.6	160.31	0.3663	8.37
Mg-0.5Ca-3.0Y	−2969.2	63.4	−156.6	276.38	0.4463	10.20

**Table 6:** Chemical composition of Mg-0.5Ca-xY alloy surface after electrochemical test.

	Chemical Elements	Mg	Ca	Y	O	Cl	Na	K
		wt.%	wt.%	wt.%	wt.%	wt.%	wt.%	wt.%
Mg-0.5Ca-0.5Y	Surface with oxides	61.6	0.4	0.4	37.6	-	-	-
	Surface without oxides	96.7	1.3	2.1	-	-	-	-
Mg-0.5Ca-1.0Y	Surface with oxides	58.4	0.7	0.5	33.2	4.0	2.4	-
	Surface without oxides	95.6	2.3	2.1	-	-	-	-
Mg-0.5Ca-1.5Y	Surface with oxides	45.9	0.7	0.9	46.3	-	5.4	0.9
	Surface without oxides	90.2	3.0	6.8	-	-	-	-
Mg-0.5Ca-2.0Y	Surface with oxides	44.1	0.8	0.9	50.1	0.4	3.8	-
	Surface without oxides	87.8	4.0	8.2	-	-	-	-
Mg-0.5Ca-3.0Y	Surface with oxides	47.7	0.5	2.8	44.8	-	1.8	2.9
	Surface without oxides	83.6	3.1	13.4	-	-	-	-

## References

[1] Schumann P., Lindhorst D., Wagner M., Schramm A., Gellrich N.C., Rucker M. (2013). Perspectives on ResorbableOsteosynthesis Materials in Cranio maxilla facial Surgery. Pathobiology.

[2] Rahim M.I., Ullah S., Mueller P. (2018). Advances and Challenges of Biodegradable Implant Materials with a Focus on Magnesium-Alloys and Bacterial Infections. Metals.

[3] Banerjee P.C., Al-Saadi S., Choudhary L., Harandi S.E., Singh R. (2019). Magnesium Implants: Prospects and Challenges. Materials.

[4] Tune D. (1991). Body-Absorbable Osteosynthesis Devices. Clin. Mater..

[5] Staiger M.P., Pietak A.M., Huadmai J., Dias G. (2006). Magnesium and its alloys as orthopedic biomaterials: A review. Biomaterials.

[6] Sandu A.V., Baltatu M.S., Nabialek M., Savin A., Vizureanu P. (2019). Characterization and Mechanical Proprieties of New TiMo Alloys Used for Medical Applications. Materials.

[7] Baltatu M.S., Tugui C.A., Perju M.C., Benchea M., Spataru M.C., Sandu A.V., Vizureanu P. (2019). Biocompatible Titanium Alloys used in Medical Applications. Rev. Chim..

[8] Han H.S., Loffredo S., Jun I., Edwards J., Kim Y.C., Seok H.K., Witte F., Mantovani D., Glyn-Jones S. (2019). Current status and outlook on the clinical translation of biodegradable metals. Mater. Today.

[9] Jahnen-Dechent W., Ketteler M. (2012). Magnesium basics. Clin. Kidney J..

[10] Al Alawi A.M., Majoni S.W., Falhammar H. (2018). Magnesium and Human Health: Perspectives and Research Directions. Int. J. Endocrinol..

[11] Ding W. (2016). Opportunities and challenges for the biodegradable magnesium alloys as next-generation biomaterials. Regen. Biomater..

[12] Atrens A., Johnston S., Shi Z., Dargusch M.S. (2018). Viewpoint—Understanding Mg corrosion in the body for biodegradable medical implants. Scr. Mater..

[13] Liu M., Uggowitzer P.J., Nagasekhar A.V., Schmutz P., Easton M., Song G.L., Atrens A. (2009). Calculated phase diagrams and the corrosion of die-cast Mg–Al alloys. Corros. Sci..

[14] Riaz U., Shabib I., Haider W. (2019). The current trends of Mg alloys in biomedical applications—A review. J. Biomed. Mater. Res. B Appl. Biomater..

[15] Liu D., Yang D., Li X., Hu S. (2019). Mechanical properties, corrosion resistance and biocompatibilities of degradable Mg-RE alloys: A review. J. Mater. Res. Technol..

[16] Angrisani N., Reifenrath J., Seitz J.M., Meyer-Lindenberg A., Monteiro W.A. (2012). Rare Earth Metals as Alloying Components in Magnesium Implants for Orthopaedic Applications. New Features on Magnesium Alloys.

[17] Hirano S., Suzuki K. (1996). Exposure, Metabolism, and Toxicity of Rare Earths and Related Compounds. Environ. Health Perspect..

[18] Rim K.T., Koo K.H., Park J.S. (2013). Toxicological Evaluations of Rare Earths and Their Health Impacts to Workers: A Literature Review. Saf. Health Work.

[19] Pagano G., Guida M., Tommasi F., Oral R. (2015). Health effects and toxicity mechanisms of rare earth elements—Knowledge gaps and research prospects. Ecotoxicol. Environ. Saf..

[20] Tekumalla S., Seetharaman S., Almajid A., Gupta M. (2015). Mechanical Properties of Magnesium-Rare Earth Alloy Systems: A Review. Metals.

[21] Peng Q., Huang Y., Zhou L., Hort N., Kainer K.U. (2010). Preparation and properties of high purity Mg–Y biomaterials. Biomaterials.

[22] Liu M., Schmutz P., Uggowitzer P., Song G., Atrens A. (2010). The influence of Y (Y) on the corrosion of Mg–Y binary alloys. Corros. Sci..

[23] Sudholz A.D., Gusieva K., Chen X.B., Muddle B., Gibson M., Birbilis N. (2011). Electrochemical behaviour and corrosion of Mg–Y alloys. Corros. Sci..

[24] Südholz A.D., Kirkland N.T., Buchheit R.G., Birbilis N. (2011). Electrochemical Properties of Intermetallic Phases and Common Impurity Elements in Magnesium Alloys. Electrochem. Solid-State Lett..

[25] Lupescu S., Munteanu C., Istrate B., Stanciu S., Cimpoesu N., Oprisan B. (2017). Microstructural Investigations on Alloy Mg-2Ca-0.2Mn-0.5Zr-1Y. IOP Conf. Ser. Mater. Sci. Eng..

[26] Ben-Hamu G., Eliezer D., Shin K.S., Cohen S. (2007). The relation between microstructure and corrosion behavior of Mg–Y–RE–Zr alloys. J. Alloys Compounds.

[27] Liu J., Bian D., Zheng Y., Chu X., Lin Y., Wang M., Lin Z., Li M., Zhang Y., Guan S. (2020). Comparative in vitro study on binary Mg-RE (Sc, Y, La, Ce, Pr, Nd, Sm, Eu, Gd, Tb, Dy, Ho, Er, Tm, Yb and Lu) alloy systems. Acta Biomater..

[28] Pravina P., Sayaji D., Avinash M. (2013). Calcium and its Role in Human Body. Int. J. Res. Pharm. Biomed. Sci..

[29] Pang X., Lin L., Tang B. (2017). Unraveling the role of Calcium ions in the mechanical properties of individual collagen fibrils. Sci. Rep..

[30] Pu F., Chen N., Xue S. (2016). Calcium intake, calcium homeostasis and health. Food Sci. Hum. Wellness.

[31] Peron M., Torgersen J., Berto F. (2017). Mg and Its Alloys for Biomedical Applications: Exploring Corrosion and Its Interplay with Mechanical Failure. Metals.

[32] Witte F., Hort N., Vogt C., Cohen S., Kainer K.U., Willumeit R., Feyerabend F. (2008). Degradable biomaterials based on magnesium corrosion. Curr. Opin. Solid State Mater. Sci..

[33] Kim W.C., Kim J.G., Lee J.Y., Seok H.K. (2008). Influence of Ca on the corrosion properties of magnesium for biomaterials. Mater. Lett..

[34] Wan Y., Xiong G., Luo H., He F., Huang Y., Zhou X. (2008). Preparation and characterization of a new biomedical magnesium–calcium alloy. Mater. Des..

[35] Li Z., Gu X., Lou S., Zheng Y. (2008). The development of binary Mg-Ca alloys for use as biodegradable materials within bone. Biomaterials.

[36] Von Der Hoh N., Bormann D., Lucas A., Denkena B., Hackenbroich C., Meyer-Lindenberg A. (2009). Influence of different surface machining treatments of magnesium-based resorbable implants on the degradation behavior in rabbits. Adv. Eng. Mater..

[37] Li Y., Li M., Hu W., Hodgson P., Wen C. (2010). Biodegradable Mg-Ca and Mg-Ca-Y alloys for Regenerative Medicine. Mater. Sci. Forum.

[38] Liu C.L., Wang Y.J., Zeng R.C., Zhang X.M., Huang W.J., Chu P.K. (2010). In vitro corrosion degradation behaviour of Mg–Ca alloy in the presence of albumin. Corros. Sci..

[39] Kirkland N., Birbilis N., Walker J., Woodfield T., Dias G., Staiger M. (2010). In-vitro dissolution of magnesium–calcium binary alloys: Clarifying the unique role of calcium additions in bioresorbable magnesium implant alloys. J. Biomed. Mater. Res. Part B Appl. Biomater..

[40] Krause A., von der Hoh N., Bormann D., Krause C., Bach F.W., Windhagen H., Meyer-Lindenberg A. (2010). Degradation behaviour and mechanical properties of magnesium implants in rabbit tibiae. J. Mater. Sci..

[41] Erdmann N., Angrisani N., Reifenrath J., Lucas A., Thorey F., Bormann D., Meyer-Lindenberg A. (2011). Biomechanical testing and degradation analysis of MgCa0.8 alloy screws: A comparative in vivo study in rabbits. Acta Biomater..

[42] Harandi S.E., Idris M.H., Jafari H. (2011). Effect of forging process on microstructure, mechanical and corrosion properties of biodegradable Mg–1Ca alloy. Mater. Des..

[43] Salahshoor M., Guo Y. (2012). Biodegradable Orthopedic Magnesium-Calcium (MgCa) Alloys, Processing, and Corrosion Performance. Materials.

[44] Rad H.R.B., Idris M.H., Kadir M.R.A., Farahany S. (2012). Microstructure analysis and corrosion behavior of biodegradable Mg–Ca implant alloys. Mater. Des..

[45] Li N., Zheng Y. (2013). Novel Magnesium Alloys Developed for Biomedical Application: A Review. J. Mater. Sci. Technol..

[46] Jeong Y.S., Kim W.J. (2014). Enhancement of mechanical properties and corrosion resistance of Mg–Ca alloys through microstructural refinement by indirect extrusion. Corros. Sci..

[47] Jeong Y.S., Kim W.J. (2015). Development of biodegradable Mg–Ca alloy sheets with enhanced strength and corrosion properties through the refinement and uniform dispersion of the Mg_2_Ca phase by high-ratio differential speed rolling. Acta Biomater..

[48] Zeng R.C., Qi W.C., Cui H.Z., Zhang F., Li S.Q., Han E.H. (2015). In vitro corrosion of as-extruded Mg–Ca alloys—The influence of Ca concentration. Corros. Sci..

[49] Bita A.I., Antoniac A., Cotrut C., Vasile E., Ciuca I., Niculescu M., Antoniac I. (2016). In Vitro Degradation and Corrosion Evaluation of Mg-Ca Alloys for Biomedical Applications. J. Optoelectron. Adv. Mater..

[50] Rau J.V., Antoniac I., Fosca M., De Bonis A., Blajan A.I., Cotrut C., Graziani V., Curcio M., Cricenti A., Niculescu M. (2016). Glass-ceramic coated Mg-Ca alloys for biomedical implant applications. Mater. Sci. Eng. C.

[51] Hunan High Broad New Material, Co.Ltd. http://www.hbnewmaterial.com/supplier-129192-master-alloy.

[52] Lupescu S., Istrate B., Munteanu C., Minciuna M.G., Focsaneanu S., Earar K. (2017). Characterization of Some Master Mg-X System (Ca, Mn, Zr, Y) Alloys Used in Medical Applications. Rev. Chim..

[53] Mansfield F., Bertocci U. (1981). Electrochemical Corrosion Testing. ASTM STP.

[54] Mosmann T. (1983). Rapid colorimetric assay for cellular growth and survival: Application to proliferation and cytotoxicity assays. J. Immunol. Methods.

[55] Vlad M.D., Valle L.J., Poeată I., Barracó M., López J., Torres R., Fernández E. (2008). Injectable iron-modified apatitic bone cement intended for kyphoplasty: Cytocompatibility study. J. Mater. Sci. Mater. Med..

[56] Vlad M.D., Valle L.J., Poeată I., López J., Torres R., Barracó M., Fernández E. (2010). Biphasic calcium sulfate dihydrate/iron-modified alpha-tricalcium phosphate bone cement for spinal applications: In vitro study. Biomed. Mater..

[57] Istrate B., Munteanu C., Lupescu S., Antoniac V.I., Sindilar E. (2018). Structural Characterization of Mg-0.5Ca-xY Biodegradable Alloys. Key Eng. Mater..

[58] Atrens A., Song G.L., Cao F., Shi Z., Bowen P.K. (2013). Advances in Mg corrosion and research suggestions. J. Magnes. Alloy..

[59] Li Z., Sun S., Chen M., Fahlman B.D., Liu D., Bi H. (2017). In vitro and in vivo corrosion, mechanical properties and biocompatibility evaluation of MgF2-coated Mg-Zn-Zr alloy as cancellous screws. Mater. Sci. Eng. C.

[60] ISO 10993-5:2009—Biological Evaluation of Medical Devices—Part 5: Tests for In Vitro Cytotoxicity. http://nhiso.com/wp-content/uploads/2018/05/ISO-10993-5-2009.pdf.

[61] Gilles R., Belkhir M., Compere P., Libioulle C., Thiry M. (1995). Effect of high osmolarity acclimation on tolerance to hyperosmotic shocks in L929 cultured cells. Tissue Cell.

[62] Mbele G.O., Deloulme J.C., Gentil B.J., Delphin C., Ferro M., Garin J., Takahashi M., Baudier J. (2002). The zinc and calcium-binding S100B interacts and co-localizes with IQGAP1 during dynamic rearrangement of cell membranes. J. Biol. Chem..

[63] Yang L., Hort N., Laipple D., Höche D., Huang Y., Kainer K.U., Willumeit R., Feyerabend F. (2013). Element distribution in the corrosion layer and cytotoxicity of alloy Mg–10Dy during in vitro biodegradation. Acta Biomater..

[64] Esmaily M., Svensson J.E., Fajardo S., Birbilis N., Frankel G.S., Virtanen S., Arrabal R., Thomas S., Johansson L.G. (2017). Fundamentals and advances in magnesium alloy corrosion. Prog. Mater. Sci..

